# The comet assay: past, present, and future

**DOI:** 10.3389/fgene.2015.00266

**Published:** 2015-08-13

**Authors:** Sabine A. S. Langie, Amaya Azqueta, Andrew R. Collins

**Affiliations:** ^1^Environmental Risk and Health Unit, Flemish Institute of Technological Research (VITO)Mol, Belgium; ^2^Department of Pharmacology and Toxicology, University of NavarraPamplona, Spain; ^3^Department of Nutrition, University of OsloOslo, Norway

**Keywords:** comet assay, single cell gel electrophoresis, genotoxicity, ecotoxicology, DNA repair, biomonitoring, standardization

The alkaline comet assay (single cell gel electrophoresis) is the most widely used method for measuring DNA damage in eukaryotic cells (Neri et al., 2015). It detects strand breaks (SBs) and alkali-labile sites at frequencies from a few hundred to several thousand breaks per cell—a biologically useful range, extending from low endogenous damage levels to the extent of damage that can be inflicted experimentally without killing cells. Digestion of the nucleoids, after lysis, with certain lesion-specific repair endonucleases allows measurement of damage other than SBs; notably, formamidopyrimidine DNA glycosylase (FPG) has been widely used to detect altered purines, which are converted to breaks by the enzyme. Recently, (Cortés-Gutiérrez et al., 2014) developed a two-dimensional Two-Tailed comet assay (TT-comet) that can differentiate between single-stranded (SSBs) and double-stranded DNA breaks (DSBs) in the same comets in sperm.

Since the first report by Ostling and Johanson (1984) the comet assay has been widely used in genotoxicity testing of chemicals, in both *in vitro* and *in vivo* models. An advantage with the latter is that cells from various tissues can be studied, in a wide variety of eukaryotic organisms. During the last 15 years, the comet assay has been extensively used in *Drosophila melanogaster* to test the genotoxicity of chemicals (Gaivão and Sierra, 2014). This approach is very useful since *Drosophila melanogaster* is a valuable model for all kinds of processes related to human health, including DNA damage responses.

The use of plants as well as a wide range of terrestrial and aquatic species in the comet assay has dramatically increased in the last decade (Costa et al., 2014; de Lapuente et al., 2015; Santos et al., 2015), particularly in environmental risk assessment (ERA). A recent validation study has indicated that the *in vitro* comet assay combined with FPG may be an effective complementary line-of-evidence in ERA even in particularly challenging natural scenarios such as estuarine environments (Costa et al., 2014).

During the past decade the production and use of nano-sized materials has significantly increased, and as a consequence so has human exposure to these types of materials. Identifying and understanding the hazards of nanomaterials (NMs) in relation to human health is not a simple matter. Not only is the chemical composition of NMs responsible for their genotoxicity, but also shape, specific surface area, size, size distribution, and zeta potential determine the effects of these materials on the genome. Although there is still a debate about the suitability of standard genotoxicity assays for studying the effects of NMs, so far the most used method in nanogenotoxicology, thanks to its robustness, versatility, and reliability, has been the comet assay (Azqueta and Dusinska, 2015). In addition to investigating the genotoxicity of radiation and various chemicals, the plant comet assay has recently also been used to study the genotoxic impact of NPs (Santos et al., 2015).

A further application of the comet assay is as a valuable experimental tool for human biomonitoring as well as in clinical studies. Collecting blood or tissues is not always feasible in all human subjects, and other sources of cells that can be collected non-invasively have been tested with the comet assay; for example, various types of epithelial cells (Rojas et al., 2014) as well as sperm (Cortés-Gutiérrez et al., 2014; Brunborg et al., 2015).

In parallel with the development of the comet assay for DNA damage measurement, assays for DNA repair—an essential element in the genotoxic cellular response—have been developed. The simplest approach to DNA repair measurement is to treat cells with a DNA-damaging agent and then to incubate them to allow repair to proceed, measuring the amount of damage remaining at intervals. An alternative, biochemical approach to assessing repair capacity was described in 1994 (Collins et al., 1994), and since then various modified versions of the assay to measure both base excision repair (BER) and nucleotide excision repair (NER) have been published (reviewed by Azqueta et al., 2014). This biochemical approach has been applied to study the effects of environment, nutrition, lifestyle, and occupation on DNA repair capacity, in addition to clinical investigations (Azqueta et al., 2014).

This alternative *in vitro* approach to DNA repair assesses the repair activity of a cell extract on a DNA substrate containing defined lesions. The comet assay is used to follow the accumulation of DNA breaks (repair intermediates) with time of incubation. Recently, Slyskova and colleagues were the first to apply the *in vitro* DNA repair assays for BER and NER successfully on human tissue samples; specifically, colorectal carcinoma biopsies (Slyskova et al., 2012, 2014).

A different kind of DNA repair assay, allowing cells embedded in the gel to repair before lysis, was recently adopted to study DNA repair kinetics in more detail; specifically, to study the regulation of BER proteins by post-transcriptional modifications (Nickson and Parsons, 2014). Yet another way to study DNA repair, at the level of specific genes, is with the comet-FISH technique, which makes use of fluorescent-labeled DNA probes that will hybridize to the single-stranded DNA in the comet tail. McAllister et al. (2014) used this method to study preferential strand break repair in bulk DNA as well as in selected regions with actively transcribed genes.

Studying the kinetics of repair of induced damage will help in our understanding of cellular responses to genotoxic chemicals. Moreover, the significance of DNA repair as a player in the (anti)carcinogenic process can be elucidated by looking at repair at the level of specific cancer target tissues. Regulation of repair—and other aspects of the cellular response to genotoxic compounds—is likely to involve epigenetic mechanisms and the comet assay has been adopted successfully to measure changes in the global DNA methylation pattern in individual cells under various growth conditions (Lewies et al., 2014).

Per cent tail DNA is recommended as the best descriptor for DNA break frequencies, as the comets referred to—and extent of damage—can easily be visualized. However, many researchers still prefer the use of tail moment (Møller et al., 2014). In fact the two descriptors are similarly influenced by assay conditions (Azqueta et al., 2011; Ersson and Möller, 2011).

Variability in the comet assay is an important issue, whether it arises from the use of different protocols, or from uncontrollable or random experimental variation. The inclusion of reference standards in all experiments is recommended, especially when a large number of samples—from a biomonitoring trial, for example—are analyzed on different occasions. Reference standards are cells with a known amount of DNA damage; either untreated cells (negative control), X-ray-exposed cells (positive control), or cells treated with photosensitizer plus light (positive control for assays including FPG-incubation), batch-prepared and frozen as aliquots. If substantial variation occurs in the standards in a run of experiments, sample results can be normalized (Collins et al., 2014). If reference standards are exchanged between laboratories, results from these laboratories can more easily be compared.

Reference standard cells are normally set in gels in parallel to sample gels. Internal standards—i.e., standard cells in the same gel as sample cells—would be ideal; but it is of course essential to be able to distinguish the two types of cell. Fish cells that are either larger or smaller in genome size compared to human cells have successfully been adopted for this purpose (Brunborg et al., 2015). These reference cells can be used in combination with a standard or calibration curve (established with cells given different doses of ionizing radiation), enabling a more precise quantification of DNA lesions expressed as a DNA break frequency rather than % tail DNA.

Statistics are an important tool in all applications of the comet assay, to check whether small differences occur by chance. Concise descriptions of statistical analysis and recommendations for tests have been published (Lovell et al., 1999; Lovell and Omori, 2008). Møller and Loft (2014) remind us that to keep the comet assay statistical analysis simple, appropriate study design and statistical power should be carefully considered when planning experiments.

As with all biological assays, data integration is crucial to interpret the comet assay results within the bigger picture. Integration of information provided by the comet assay with other DNA-damage indicators and cellular responses (e.g., oxidative stress, cell division, or cell death) has been applied both in ERA (Costa et al., 2014; Santos et al., 2015) as well as human (biomonitoring) studies (e.g., Langie et al., 2010; Slyskova et al., 2012). Also including “omics” data will aid in unraveling the mode of action of genotoxic compounds (Slyskova et al., 2012, 2014; Santos et al., 2015)—though it is worth pointing out that several studies have shown that phenotypic measures of DNA repair do not necessarily correlate with genomic or transcriptomic data (Collins et al., 2012; Slyskova et al., 2012, 2014); the different approaches should be regarded as complementary.

Even after three decades of development and modification, the comet assay is still a rather simple, versatile but labor-intensive assay. Various high throughput modifications of the assay were recently reviewed (Brunborg et al., 2014). Both *in vivo* and *in vitro* applications would gain great advantage from further improvements in efficiency, standardization of protocol, and throughput. Automation and miniaturization are common strategies in many areas of biology, allowing orders-of-magnitude changes in the numbers of samples analyzed per experiment, reducing subjective bias, and enhancing reproducibility.

So—what can we hope for in the next 30 years? Acceptance of the *in vitro* comet assay for genotoxicity testing, inexpensive automated comet scoring to save researchers from interminable microscope viewing, protocol standardization (perhaps) and reliable internal reference standards, more human biomonitoring studies of DNA repair (accepting that phenotypic assays have an important place alongside genomics and transcriptomics), environmental monitoring using a variety of animal and plant species; and many more unpredictable developments and applications.

## Conflict of interest statement

The authors declare that the research was conducted in the absence of any commercial or financial relationships that could be construed as a potential conflict of interest.

## References

[B1] Azqueta A. Dusinska M. (2015). The use of the comet assay for the evaluation of the genotoxicity of nanomaterials. Front. Genet. 6:239. 10.3389/fgene.2015.00239 26217380 PMC4498100

[B2] Azqueta A. Gutzkow K. B. Brunborg G. Collins A. R. (2011). Towards a more reliable comet assay: optimising agarose concentration, unwinding time and electrophoresis conditions. Mutat. Res. 724, 41–45. 10.1016/j.mrgentox.2011.05.010 21645630

[B3] Azqueta A. Slyskova J. Langie S. A. O'Neill Gaivão I. Collins A. (2014). Comet assay to measure DNA repair: approach and applications. Front. Genet. 5:288. 10.3389/fgene.2014.00288 25202323 PMC4142706

[B4] Brunborg G. Collins A. Graupner A. Gutzkow K. B. Olsen A.-K. (2015). Reference cells and ploidy in the comet assay. Front. Genet. 6:61. 10.3389/fgene.2015.00061 25774164 PMC4343028

[B5] Brunborg G. Jackson P. Shaposhnikov S. Dahl H. Azqueta A. Collins A. R.. (2014). High throughput sample processing and automated scoring. Front. Genet. 5:373. 10.3389/fgene.2014.00373 25389434 PMC4211552

[B6] Collins A. R. Azqueta A. Langie S. A. S. (2012). Effects of micronutrients on DNA repair. Eur. J. Nutr. 51, 261–279. 10.1007/s00394-012-0318-4 22362552

[B7] Collins A. R. El Yamani N. Lorenzo Y. Shaposhnikov S. Brunborg G. Azqueta A. (2014). Controlling variation in the comet assay. Front. Genet. 5:359. 10.3389/fgene.2014.00359 25368630 PMC4202776

[B8] Collins A. R. Fleming I. M. Gedik C. M. (1994). *In vitro* repair of oxidative and ultraviolet-induced DNA damage in supercoiled nucleoid DNA by human cell extract. Biochim. Biophys. Acta. 1219, 724–727. 10.1016/0167-4781(94)90236-4 7948034

[B9] Cortés-Gutiérrez E. I. López-Fernández C. Fernández J. L. Dávila-Rodríguez M. I. Johnston S. D. Gosálvez J. (2014). Interpreting sperm DNA damage in a diverse range of mammalian sperm by means of the two-tailed comet assay. Front. Genet. 5:404. 10.3389/fgene.2014.00404 25505901 PMC4245925

[B10] Costa P. M. Pinto M. Vicente A. M. Gonçalves C. Rodrigo A. P. Louro H.. (2014). An integrative assessment to determine the genotoxic hazard of estuarine sediments: combining cell and whole-organism responses. Front. Genet. 5:437. 10.3389/fgene.2014.00437 25540652 PMC4261831

[B11] de Lapuente J. Lourenço J. Mendo S. A. Borràs M. Martins M. G. Costa P. M.. (2015). The Comet Assay and its applications in the field of ecotoxicology: a mature tool that continues to expand its perspectives. Front. Genet. 6:180. 10.3389/fgene.2015.00180 26089833 PMC4454841

[B12] Ersson C. Möller L. (2011). The effects on DNA migration of altering parameters in the comet assay protocol such as agarose density, electrophoresis conditions and durations of the enzyme or the alkaline treatments. Mutagenesis 26, 689–695. 10.1093/mutage/ger034 21778357

[B13] Gaivão I. Sierra L. M. (2014). Drosophila comet assay: insights, uses, and future perspectives. Front. Genet. 5:304. 10.3389/fgene.2014.00304 25221574 PMC4148904

[B14] Langie S. A. Wilms L. C. Hämäläinen S. Kleinjans J. C. Godschalk R. W. van Schooten F. J. (2010). Modulation of nucleotide excision repair in human lymphocytes by genetic and dietary factors. Br. J. Nutr. 103, 490–501. 10.1017/S0007114509992066 19878615

[B15] Lewies A. Van Dyk E. Wentzel J. F. Pretorius P. J. (2014). Using a medium-throughput comet assay to evaluate the global DNA methylation status of single cells. Front. Genet. 5:215. 10.3389/fgene.2014.00215 25071840 PMC4083187

[B16] Lovell D. P. Omori T. (2008). Statistical issues in the use of the comet assay. Mutagenesis 23, 171–182. 10.1093/mutage/gen015 18385511

[B17] Lovell D. P. Thomas G. Dubow R. (1999). Issues related to the experimental design and subsequent statistical analysis of *in vivo* and *in vitro* comet studies. Teratog. Carcinog. Mutagen. 19, 109–119. 10332808

[B18] McAllister K. A. Yasseen A. A. McKerr G. Downes C. S. McKelvey-Martin V. J. (2014). FISH comets show that the salvage enzyme TK1 contributes to gene-specific DNA repair. Front. Genet. 5:233. 10.3389/fgene.2014.00233 25152750 PMC4126492

[B19] Møller P. Loft S. (2014). Statistical analysis of comet assay results. Front. Genet. 5:292. 10.3389/fgene.2014.00292 25221569 PMC4145634

[B20] Møller P. Loft S. Ersson C. Koppen G. Dusinska M. Collins A. R. (2014). On the search for an intelligible comet assay descriptor. Front. Genet. 5:217. 10.3389/fgene.2014.00217 25101109 PMC4101262

[B21] Neri M. Milazzo D. Ugolini D. Milic M. Campolongo A. Pasqualetti P.. (2015). Worldwide interest in the comet assay: a bibliometric study. Mutagenesis 30, 155–163. 10.1093/mutage/geu061 25527738

[B22] Nickson C. M. Parsons J. L. (2014). Monitoring regulation of DNA repair activities of cultured cells in-gel using the comet assay. Front. Genet. 5:232. 10.3389/fgene.2014.00232 25076968 PMC4100063

[B23] Ostling O. Johanson K. J. (1984). Microelectrophoretic study of radi¬ation-induced DNA damages in individual mammalian cells. Biochem. Biophys. Res. Commun. 123, 291–298. 10.1016/0006-291X(84)90411-X 6477583

[B24] Rojas E. Lorenzo Y. Haug K. Nicolaissen B. Valverde M. (2014). Epithelial cells as alternative human biomatrices for comet assay. Front. Genet. 5:386. 10.3389/fgene.2014.00386 25506353 PMC4246922

[B25] Santos C. L. V. Pourrut B. Ferreira de Oliveira J. M. P. (2015). The use of comet assay in plant toxicology: recent advances. Front. Genet. 6:216. 10.3389/fgene.2015.00216 26175750 PMC4485349

[B26] Slyskova J. Korenkova V. Collins A. R. Prochazka P. Vodickova L. Svec J.. (2012). Functional, genetic, and epigenetic aspects of base and nucleotide excision repair in colorectal carcinomas. Clin. Cancer Res. 18, 5878–5887. 10.1158/1078-0432.CCR-12-1380 22966016

[B27] Slyskova J. Langie S. A. S. Collins A. R. Vodicka P. (2014). Functional evaluation of DNA repair in human biopsies and their relation to other cellular biomarkers. Front. Genet. 5:116. 10.3389/fgene.2014.00116 24904630 PMC4033188

